# Use of the *de novo* transcriptome analysis of silver-leaf nightshade (*Solanum elaeagnifolium*) to identify gene expression changes associated with wounding and terpene biosynthesis

**DOI:** 10.1186/s12864-015-1738-3

**Published:** 2015-07-07

**Authors:** Aphrodite Tsaballa, Alexandros Nikolaidis, Foteini Trikka, Codruta Ignea, Sotirios C. Kampranis, Antonios M. Makris, Anagnostis Argiriou

**Affiliations:** Institute of Applied Biosciences, Center for Research and Technology Hellas (CERTH), P.O. Box 60361, Thessaloniki, 57001 Greece; Department of Biochemistry, School of Medicine, University of Crete, P.O. Box 2208, Heraklion, 71003 Greece

## Abstract

**Background:**

*Solanum elaeagnifolium*, an invasive weed of the Solanaceae family, is poorly studied although it poses a significant threat to crops. Here the analysis of the transcriptome of *S. elaeagnifolium* is presented, as a means to explore the biology of this species and to identify genes related to its adaptation to environmental stress. One of the basic mechanisms by which plants respond to environmental stress is through the synthesis of specific secondary metabolites that protect the plant from herbivores and microorganisms, or serve as signaling molecules. One important such group of secondary metabolites are terpenes.

**Results:**

By next-generation sequencing, the flower/leaf transcriptome of *S. elaeagnifolium* was sequenced and *de novo* assembled into 75,618 unigenes. Among the unigenes identified, several corresponded to genes involved in terpene biosynthesis; these included *terpene synthases* (*TPSs*) and genes of the mevalonate (MVA) and the methylerythritol phosphate (MEP) pathways. Functional characterization of two of the *TPSs* showed that one produced the sesquiterpene (*E*)-caryophyllene and the second produced the monoterpene camphene. Analysis of wounded *S. elaeagnifolium* leaves has shown significant increase of the concentration of (*E*)-caryophyllene and geranyl linalool, two terpenes implicated in stress responses. The increased production of (*E*)-caryophyllene was matched to the induced expression of the corresponding *TPS* gene. Wounding also led to the increased expression of the putative *1-deoxy-D-xylulose-5-phosphate synthase 2* (*DXS2*) gene, a key enzyme of the MEP pathway, corroborating the overall increased output of terpene biosynthesis.

**Conclusions:**

The reported *S. elaeagnifolium de novo* transcriptome provides a valuable sequence database that could facilitate study of this invasive weed and contribute to our understanding of the highly diverse Solanaceae family. Analysis of genes and pathways involved in the plant’s interaction with the environment will help to elucidate the mechanisms that underly the intricate features of this unique *Solanum* species.

**Electronic supplementary material:**

The online version of this article (doi:10.1186/s12864-015-1738-3) contains supplementary material, which is available to authorized users.

## Background

*Solanum elaeagnifolium* (common name: silver-leaf nightshade) is a perennial weed of the family Solanaceae, native to north Mexico and south USA [1], now extended to nearly all the Mediterranean [2]. The weed constitutes a big threat to major crops such as cotton, wheat and tomato, while it endangers city parks in metropolitan areas. Its highly invasive nature is due to its fine adaptation to diverse environmental and soil conditions (especially drought), and its reproductive mode which includes both sexual reproduction by seeds and asexual reproduction by underground regenerating buds [3, 4]. *S. elaeagnifolium* plants are also hosts to several dangerous plant viruses like potato virus Y (PVY) [5] and tomato yellow leaf curl virus (TYLCV) [6].

Although *S. elaeagnifolium* fruit is toxic to many animals [7], whole plant extracts were recently shown to exhibit analgesic, anti-inflammatory, antioxidant and hepatoprotective activities [8]. Many of these functions were attributed to the high amount of phytosterols, which amounted to more than 11 % of the plant’s extract [8]. Sterols belong to the large family of plant terpenes whose biosynthesis in plants is extremely important due to their role as phytohormones and photosynthesis pigments but more importantly as mediators of plant’s interaction with a variety of biotic and abiotic factors. Tomato breeding has been focused lately in improving the biosynthetic pathways that lead to the production of terpenes in an effort to increase herbivore resistance [9]. Wild *Solanum* species are considered a valuable source of genetic variability towards this goal [9]. Plant terpenes are produced by prenyl diphosphates, such as dimethylallyl diphosphate (DMAPP), geranyl diphosphate (GPP), farnesyl diphosphate (FPP), and geranylgeranyl diphosphate (GGPP), via two pathways, the MVA pathway and the MEP pathway [10]. Sesquiterpenes (C_15_) and triterpenes (C_30_) are produced by the cytosolic MVA pathway while monoterpenes (C_10_) and diterpenes (C_20_) are produced by the plastidial MEP pathway. However, in *Solanum* species the production of many monoterpenes and sesquiterpenes rather derives from GPP, (*Z,Z*)-FPP and neryl diphosphate (NPP) located in the plastids [11–13]. Prenyl diphosphates are the substrates on which the enzymes responsible for the production of terpenes act. The specific enzymes are TPSs and expression of their coding genes is frequently induced in response to biotic and abiotic stress [14]. Plant terpenes are implicated in a variety of plant processes such as the formation of plant hormones gibberellins (GA) and abscisic acid (ABA), the production of phytoalexins, allelopathic substances [14] and substances that attract pollinators or repel herbivores [15]. Tomato terpenes, which have been studied extensively, are abundant in the glandular trichomes of leaves, stems, young fruits and flower parts.

Although *S. elaeagnifolium* is a species that gained significant agronomic and scientific attention, only 169 expressed sequence tags (ESTs) sequences exist in GenBank. At the molecular level, it was only recently that specific EST- simple sequence repeat (SSR) molecular markers were developed and used for estimating the genetic diversity of *S. elaeagnifolium* natural accessions collected from nine sites of southeastern Australia [16]. SSR markers from other *Solanum* species have been used before for estimating the genetic variability of *S. elaeagnifolium* populations [17]. Transcriptome analyses of species such as tomato (*Solanum lycopersicum*), pepper (*Capsicum annuum*) and tobacco (*Nicotiana tabacum*) have shown that a high level of sequence conservation exists among Solanaceae [18].

In this study aiming to obtain transcriptome sequences, next-generation sequencing was performed in a pool of mRNAs isolated from *S. elaeagnifolium* leaves and flowers. By the use of computational methods transcript abundance was estimated. To assess aspects of stress resistance in *S. elaeagnifolium*, terpene biosynthesis associated with stresses and the plant’s response to leaf wounding was examined. In this context, two terpene synthases were isolated and characterized in yeast, a monoterpene synthase mostly producing camphene and lesser amounts of β-myrcene and limonene, and a sesquiterpene synthase producing mostly caryophyllene and lesser α-humulene. Leaf wounding experiments showed both transcriptional induction and caryophyllene production in wounded tissues.

## Results

### High-throughput sequencing and transcriptome assembly

The sequencing output of *S. elaeagnifolium* flowers and leaves mRNA is shown in Table 1. Clean reads were assembled into contigs using Trinity [19]. Then the reads were mapped back to contigs. An amount of 138,604 contigs were generated with a mean length of 385 nucleotides (nt) (N50 824 nt). Contigs were re-assembled into 75,618 unigenes with mean length of 1,082 nt (N50 1,778 nt). For a detailed graph of contigs and unigenes length see Additional file 1: Figure S1. A total of 33,893 clusters (prefix cl) were created from unigenes while 41,725 unigenes remained as singletons (prefix unigene).

**Table 1:** Results of Illumina sequencing

Sample	Total raw reads	Total clean reads	Total clean nucleotides	Q20 percentage	N percentage	GC percentage
*S. elaeagnifolium* leaves and flowers	54.58 MB	51.23 MB	4.61 GB	97.17 %	0.01 %	42.23 %

Statistics of mRNA sequencing of *S. elaeagnifolium* leaves and flowers. Q stands for Phred quality score indicating base calling accuracy (Q20 percentage = % bases of Q20). N percentage is the occurrence of N (any nucleotide) in the sample

### Functional characterization of unigenes

**Fig. 1:**
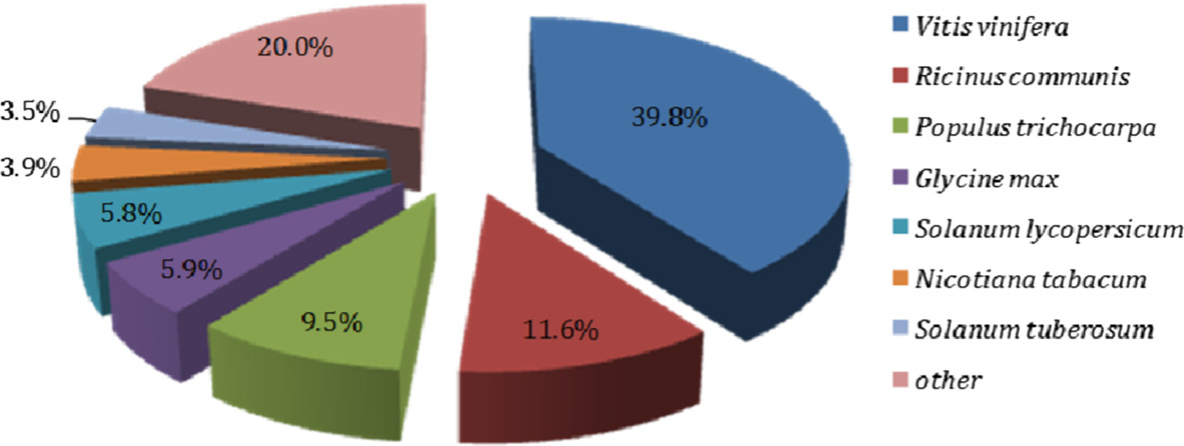
Percentage distribution of *S. elaeagnifolium* sequences based on their BLAST similarity with NR database. The percentages of *S. elaeagnifolium* unigenes similar to sequences deposited in NR database, from various plant species, are referred

Based on basic local alignment search tool (BLAST) searches in the non-redundant (NR) database at NCBI (download 14 April 2014), the majority of *S. elaeagnifolium* unigenes (39.8 %) shares similarity with grape sequences while less than 6 % of sequences shares similarity with other Solanaceae sequences (Fig. 1). Most unigenes (66.3 %) show significant similarity above 60 % with NR entries from which 25.4 % exceeds 80 % similarity.

All unigenes were employed in Blastx searches against the NR, Swiss-Prot, Kyoto encyclopedia of genes and genomes (KEGG), gene ontology (GO) and clusters of orthologous groups (COG) databases with an e-value of 10^−5^. The information obtained was used to extract coding DNA sequence (CDS) from unigenes and translate them into amino acid sequences. The CDS of unigenes that had no Blastx hit was predicted based on the ESTScan results and their translation into amino acid sequences; these unigenes were singificantly less than those whose prediction of CDS was based on BLAST results. The predicted CDS length (predicted from both BLAST results and ESTScan) was less than 500 nt with the majority of CDS being approximately 300 nt size.

Among the 36,504 unigenes with one at least GO-term given, 40.6 % were annotated in the biological process, 38.9 % in the cellular component and 20.4 % in the molecular function (for the detailed classification of the unigenes in the individual GO-terms of the three GO ontology domains see Additional file 2: Figure S2). Furthermore 19,911 unigenes were classified in 25 COG functional categories. For most of the unigenes only a general function prediction is possible (6,606 unigenes) while the next most abundant categories are transcription (3,333 unigenes), replication, recombination and repair (3,192 unigenes) and signal transduction mechanisms (2,905 unigenes) (for the detailed classification of *S. elaeagnifolium* unigenes according to COG see Additional file 3: Figure S3).

### Expression of unigenes

Transcript abundances were estimated for *S. elaeagnifolium* unigenes by the RSEM software [20]. The complete list of the 20 most expressed genes in *S. elaeagnifolium* leaves and flowers is presented in Table 2. The comparison of *S. elaeagnifolium* leaf and flower transcriptome expression results of the present study, produced by RSEM analysis, with other Solanaceae transcriptomes has shown that the majority of the most abundant transcripts are common inside the family. For instance, the most abundant transcripts in *S. elaeagnifolium* leaves and flowers encode a putative subunit of the ribulose-1,5-bisphosphate carboxylase/oxygenase (Rubisco) protein, a Rubisco activase, putative lipid-transfer proteins, proteins involved in chlorophyl binding and photosystem I and II, a S-adenosylmethionine decarboxylase (SAMDC) etc. Most of these transcripts are universally identified as strongly expressed in *Solanum* databases (tomato, potato transcriptomes) [21, 22]. Transcripts strongly expressed that code for metallothioneins (MTs) are also found. MTs are proteins that bind metal ions and are classified in four classes/types depending on the amount and the arrangement of their cysteine-rich domains [23]. MT proteins are known to respond to metal presence but also may play a role in reactive oxygen species detoxification [for a review on MT roles see [24]. An additional abundant *S. elaeagnifolium* transcript encodes a putative plastidic aldolase, an enzyme [Enzyme Commission number (EC): 4.1.2.13] that catalyzes the formation of d-glyceraldehyde-3-phosphate (GAP) and dihydroxyacetone phosphate (DHAP) from fructose-1,6-bisphosphate (FBP). Two plastidic aldolases from species *N. paniculata* were found in the leaves of this plant known for its tolerance in low-water conditions [25]. *S. elaeagnifolium* gene shares a high homology (>90 %) with these genes both responding also to salt stress [25].

**Table 2:** The 20 most expressed genes in *S.elaeagnifolium* leaves and flowers

*S.elaeagnifolium* transcript	Length (nt)	Annotation (species)	FPKM
cl588	962	Rubisco small subunit (SOLTU)	7,060.3
cl3504	1,874	Rubisco activase (chloroplast) (CAPAN)	6,226.3
unigene21118	774	PR4 leaf-precursor (SOLLC)	5,695.6
unigene17928	861	Non-specific lipid-transfer protein 2-like (SOLLC)	4,544.6
cl644	1,089	Chlorophyll a-b binding protein 3C chloroplastic-like (SOLTU)	3,960.1
cl6611	921	Photosystem II 10 kDa polypeptide chloroplastic (SOLTU)	3,372.1
unigene12950	946	Non-specific lipid-transfer protein 1-like (SOLTU)	3,007.7
cl8797	1,470	Plastidic aldolase (SOLTU)	2,583.8
unigene17653	1,550	Glyceraldehyde-3-phosphate dehydrogenase A, chloroplastic-like (SOLTU)	2,355.9
unigene680	711	Cell wall protein precursor (SOLLC)	2,343.6
cl9787_2	602	HT-protein (SOLPE)	2,243.7
unigene11345	861	Ferredoxin-1, chloroplastic-like (SOLTU)	1,916.8
unigene22300	602	Metallothionein-like protein (CAPCH)	1,870.0
cl7445	1,732	Peroxisomal (S)-2-hydroxy-acid oxidase GLO1 (SOLLC)	1,573.3
cl9785	858	PR1 precursor/PR1a (SOLLC)	1,493.1
cl10244	1,884	SAMDC (SOLTU)	1,433.5
unigene18624	790	Photosystem I reaction center V (chloroplast) (SOLTU)	1,374.2
unigene7695	730	Photosystem II reaction center W protein, chloroplastic-like (SOLTU)	1,359.1
unigene22352	1,690	Phosphoribulokinase, chloroplastic-like (SOLTU)	1,332.0
unigene12941	480	Metallothionein (SOLNI)	1,311.0

The annotation (third column) is based on the annotation of the top hit produced by Blastx searches at the NCBI protein database. All top hits are Solanaceae proteins (species abbreviations by Uniprot). FPKM (fragments per kilobase of exon per million fragments mapped) values produced by RSEM software (fourth column)

Third in abundance is a *S. elaeagnifolium* transcript (unigene21118) that presents 84 % homology to a tomato pathogenesis-related (PR) protein [iTAG v2.3: Solyc09g007010]. A similar PR protein is produced by another abundant transcript (cl9785) that is highly similar to a tomato gene [Solyc01g106620] annotated as *PR1a* gene. Both transcripts are significantly higher expressed in *S. elaeagnifolium* than their corresponding tomato and potato putative orthologs. The potato ortholog *PR1* gene [GenBank: AJ250136.1] was isolated from *P. infestans* infected leaves and is induced significantly under pathogen and elicitor attack although it is expressed under normal conditions as well. The second *S. elaeagnifolium* transcript, cl9785, is highly similar to tomato *PR1* precursor [NCBI: NP_001234358] that is not expressed at all in tomato flowers or leaves while the corresponding potato gene, a *PR1-like* gene [Potato genomics resource: PGSC0003DMT400013094] has low expression in potato flowers and leaves. Cl9785 deduced protein sequence shares 83 % identity with pepper PR1 precursor protein that was found to be induced under bacteria infection and possibly linked with the stimulation of ethylene synthesis [26].

Finally, one more highly expressed transcript, cl9787_2, shares significant similarity with a *HT-B* gene from *S. peruvianum*, a gene involved in the self-incompatibility of wild *Solanum* genera and is not expressed in self compatible species like *S. lycopersicum* [27]. The strong expression of the gene in *S. elaeagnifolium* provides molecular evidence for the outcrossing of the species, common in wild *Solanums. S. elaeagnifolium* has another probable *HT* gene (cl9787_1) that is also expressed in flowers and leaves but lower than cl9787_2.

### Identification of genes involved in terpene biosynthesis in *S. elaeagnifolium*

Plants use a number of secondary metabolites to cope with their abiotic and biotic environment and terpenes lie in the first line of plant defence against the risks posed. Not only terpenes are responsible for the biosynthesis of necessary hormones that facilitate plant responses, but oxidative and thermal stresses are also alleviated by terpene production [28]. Furthermore, some monoterpenes have been implicated in allelopathic effects [29]. Because of the importance of plant terpenes in a plethora of biological processes related to stress responses and since *S. elaeagnifolium* is a resiliant species that grows even on degraded soils, emphasis was laid on this group of secondary metabolites.

Employing BLAST suite of programs on *S. elaeagnifolium* unigenes, genes of the MVA and MEP pathways likely to participate in the biosynthesis of terpene precursors, as long as *TPS* genes were identified. The complete list of the putative genes involved in the MVA and MEP pathways is included in Table 3. Genes for key enzymes, such as 3-hydroxy-3-methylglutaryl-coenzyme A reductase (HMGR) [EC: 1.1.1.34] and DXS [EC: 2.2.1.7] have (as in tomato) multiple paralogues that are all expressed significantly in leaves and flowers. Putative *S. elaeagnifolium acetoacetyl CoA thiolase* (*AACT*) [Solyc07g045350], *HMGR1* [Solyc02g082260] and *2-C-methyl-d-erythritol 4-phosphate cytidylyl-transferase* (*MCT*) [Solyc01g102820] genes are present in the transcriptome as multiple alleles. Also genes involved in prenyl diphosphate synthesis, such as *farnesyl pyrophosphate synthase* (*FPPS*) and *geranylgeranyl pyrophosphate synthase* (*GGPPS*), also have many paralogues. Finally, several putative *cis-prenyltransferase* genes (*CPT*) believed to be involved in the biosynthesis of long-chain polyisoprenoids were also identified in *S. elaeagnifolium*. Two of them (cl6054.contig2 and cl6054.contig4) have homology to CPT5. For the complete list of the putative *S. elaeagnifolium TPS* genes identified in leaves and flowers, see table in Additional file 4: Table S1.

**Table 3:** The *S. elaeagnifolium* putative orthologs of tomato genes involved in the MVA, MEP pathways, the prenyl phosphate metabolism and the biosynthesis of polyisoprenoids

Tomato/Potato Gene ID	Leaf	Flower	*S.elaeagnifolium* putative ortholog	FPKM
AACT Solyc05g017760	19.5	22.7	cl7898.contig1 + 2	11.3
AACT Solyc07g045350	3.3	13.4	**cl7898.contig3**	53.2
			**cl7898.contig4**	14.4
HMGS Solyc08g007790	18.5	14.2	cl4122.contig2 + unigene12243	5.2
			+unigene15765 + unigene15766	
HMGS Solyc08g080170	25.3	33.3	cl4122.contig3	41.7
HMGR1 Solyc02g082260	11.8	86.6	**cl1634.contig1**	25.6
			**cl1634.contig2**	58.1
			cl1634.contig4	38.1
HMGR2 Solyc02g038740 (HMG2)/PGSC0003DMP400006164	1.1	49.8	**cl1634.contig3 (90 %)**	285.8
HMGR Solyc03g032010	15.3	44.9	unigene23036 + unigene23038	16.4
MVK Solyc01g098840	12.7	9.6	**unigene21441**	31.8
PMK Solyc08g076140	6.7	10.9	cl9083.contig2 + unigene25409	7.4
MVD Solyc11g007020	14.7	25.9	cl9530.contig2	40.4
MVD Solyc04g009650	5.6	13.5	unigene12676 + unigene32872	1.8
IDI Solyc04g056390	61.5	193.5	**cl5610.contig1**	98.5
IDI Solyc05g055760	16.0	21.7	**unigene22921**	37.5
FPS1 Solyc12g015860	51.0	51.9	**cl2768.contig1 (91 %)**	117.9
			unigene28826 (88 %)	24.9
FPS Solyc10g005810	3.5	4.7	unigene6739	51.5
DXS Solyc01g067890	142.3	116.4	unigene22809 (96.7 %)	91.1
DXS2 Solyc11g010850	27.6	459.2	unigene2314 (92 %)	146.6
DXR Solyc03g114340	126.2	382.9	**unigene24922**	107.5
MCT Solyc01g102820 (CMS)	19.3	31.9	cl4081.contig1	0.8
			cl4081.contig2	1.3
			cl4081.contig3	0
			cl4081.contig4	20.4
CMK Solyc01g009010 (ISPE)	25.5	38.9	cl7176.contig1	19.0
MDS Solyc08g081570	56.6	30.5	unigene15434	63.2
HDS Solyc11g069380 (GcpE)	135.3	541.6	**unigene21842**	220.3
HDR Solyc01g109300	218.8	204.9	**unigene23731**	386.2
GGPS Solyc02g085700	61.3	163.4	**unigene9264**	284.8
GGPS Solyc09g008920	80.3	124.6	unigene20920	48.9
GGPS2 Solyc04g079960	18.3	69.9	**unigene23673**	18.2
GGPS Solyc09g008920	80.3	124.6	unigene20920	48.9
GPS Solyc08g023470	23.9	20.7	unigene17628	2.6
CPT3 Solyc03g025560	8.5	8.6	cl6749.contig1	8.0
CPT4 Solyc10g085150	5.4	5.9	cl6054.contig2	10.7
CPT5 Solyc10g085140	24.1	120.9	cl6054.contig4	159.7
CPT7 Solyc06g076920	15.8	2.2	unigene38545	0.3

The tomato leaf and flower RPKMs are provided by tomato functional genomics database (TFGD) (http://ted.bti.cornell.edu/) based on cv. “Heinz” RNA-sequencing (RNA-seq) data. *S. elaeagnifolium* unigenes in bold have full length similarity (%) to tomato genes. FPKM values by RSEM software

Three *TPS* genes (cl7653, cl1310 and cl9841), the putative *HMGR1* gene (cl1634), and the *DXS2* gene (unigene2314) were selected for further analysis. The specific *TPS* genes were selected for study because they are putatively involved in the production of all three major classes of terpenes, mono-, di- and sesqui- terpenes. *HMGR* and *DXS* code for critical enzymes of the two terpene biosynthesis pathways [30]. According to BLAST results, *HMGR1* has three alleles; three contigs that belong to the cluster cl1634. Cl1634 contigs 1, 2 and 4 have 92, 95 and 91 % similarity with tomato *HMGR1* and all have significant FPKM values in the pooled mRNA from leaves and flowers (Table 3). On the contrary, unigene2314 is the only *S. elaeagnifolium* sequence that has high homology (92 %) with a tomato characterized *DXS* gene (Table 3).

For the putative *TPS* genes, transcript cl7653 is a cluster of eight sequences. Analysis of the sequences included in cl7653 indicates alternative splicing events taking place during the transcription of the corresponding gene. The different transcripts differ in three regions: in the first, four sequences have a 122 nt insertion, in the second, four sequences have a 94 nt insertion and in the last, four sequences have a 88 nt insertion. None of the inserted sequences has an open reading frame (ORF) indicative of functional proteins. Only one transcript/sequence of 2,143 nt contains a 1,653 nt putative CDS sequence that codes for a full 550 amino acid protein. The sequence, named hereafter cl7653, is the one with the highest FPKM value in flowers and leaves (see table in Additional file 4: Table S1) while the other seven transcripts/sequences have lowest FPKM values.

**Fig. 2:**
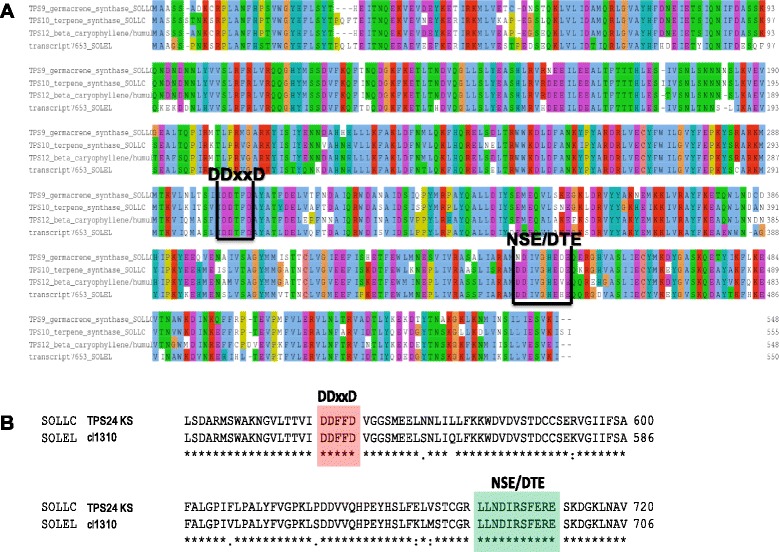
Alignment of *S. elaeagnifolium* deduced amino acid sequences with their related tomato proteins. **a** Tomato TPS9, TPS10 and TPS12 and the cl7653 predicted protein alignment. TPS9 (also called SST1) and TPS12 (also called CAHS) are closely related. Transcript cl7653 from *S. elaeagnifolium* resembles both and TPS10. The motif DDxxD, engaged in cofactor binding is fully conserved in the four proteins. The NSE/DTE motif is present as (N,D)DIVGHE(D,V,H)E following its general form (N, D)D(L, I, V)X(S, T)XXXE [61]; the fifth amino acid is a glycine (G) instead of serine (S) or threonine (T). The alignment was created by ClustalW and edited with Jalview. Amino acids that share the same coloring have similar biochemical properties. **b** Tomato TPS24 and cl1310 predicted protein alignment. The parts of the alignment depicted contain the motifs DDxxD (highlighted in red) and NSE/DTE (highlighted in green)

The alignment of cl7653 predicted protein sequence with closely related tomato proteins TPS9-sesquiterpene synthase 1 [NCBI: NP_001234481], TPS10 and TPS12 (also known as caryophyllene/α-humulene synthase - CAHS) [GenBank: AEP82783] shows a high conservation of amino acids throughout their length (Fig. 2a). TPS9 and TPS12 are known and characterized sesquiterpene synthases. The deduced cl7653 protein contains the DDxxD and NSE/DTE motifs (both boxed in Fig. 2a) that characterize TPS proteins.

The second putative *TPS* gene in study, cl1310, is a cluster of 25 sequences. However according to RSEM analysis only two of them are expressed above a FPKM threshold of 3. The two sequences differ only in a 131 nt insertion, indicating that one could correspond to an incompletely spliced transcript. The sequence of 2,801 nt that contains no intron sequence and has the highest expression in flowers and leaves, was analyzed and annotated hereafter as cl1310. Cl1310 possess a predicted CDS of 2,414 nt that codes for a 897 amino acids protein. The predicted protein shares 89 % similarity with the predicted *ent*-kaurene synthase (KS) protein from potato [NCBI: XP_006346019], 86 % similarity with tomato TPS24-KS protein [GenBank: AEP82778] [EC: 4.2.3.19] and 83 % similarity with *N. attenuata* KS protein [GenBank: AFA35954]. The alignment of tomato TPS24-KS protein with the predicted cl1310 amino acid sequence showed that the *S. elaeagnifolium* protein also contains the aspartate-rich DDxxD and NSE/DTE motifs both identical to tomato TPS24-KS corresponding motifs (Fig. 2b).

The third *TPS* gene identified, transcript cl9841 is a cluster of 9 sequences but only one is expressed in leaves and flowers; it contains a 1,824 nt putative CDS sequence that encodes a 607 amino acid full protein. The protein shares 78 % similarity with tomato TPS3 protein, a monoterpene camphene/tricyclene synthase [GenBank: AEM05853] and a putative camphene/tricyclene synthase from potato [NCBI: XP_006351730].

### Functional characterization of *S. elaeagnifolium* putative *TPS* genes in yeast cells

**Fig. 3:**
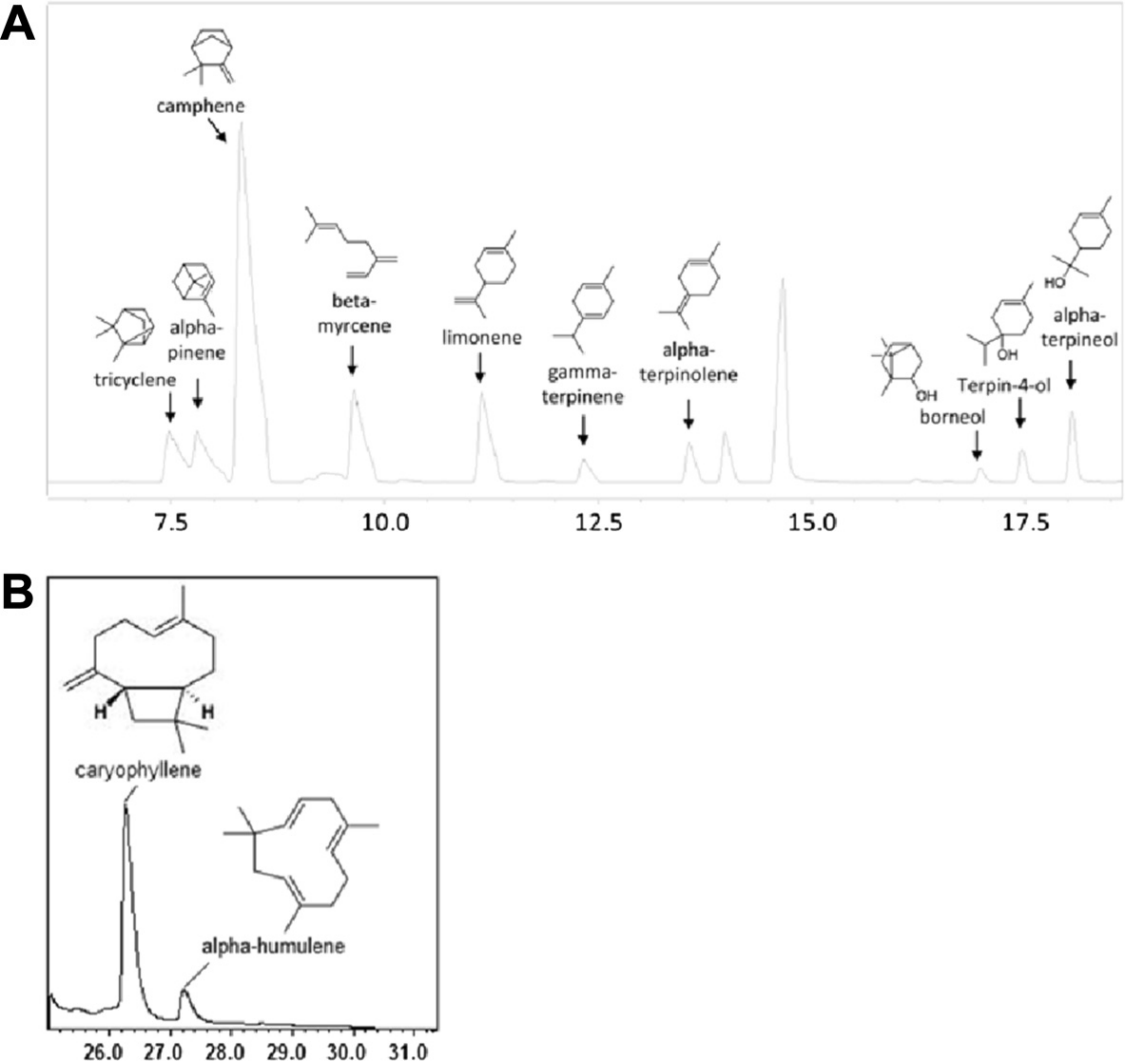
GC/MS chromatograms. **a** GC/MS chromatogram with the substances produced by cl9841 expressed in yeast. **b** GC/MS chromatogram of the dodecane phase of yeast culture. The major product seems to be caryophyllene (26.26 min) as its peak area percentage reflexes a higher amount compared to α-humulene (27.23 min) which is also produced by yeast

The yeast strain AM94 [31] was used to transform cl9841 putative monoterpene synthase together with the ERG20 (F96W-N127W) variant which shifts production towards GPP substrate [32]. For the expression of cl1310, the gene was co-expressed in AM238 cells together with copalyl diphosphate synthase from *Salvia pomifera* and a variant of yeast *ERG20* (F96C) producing GGPP. For the characterization of the putative sesquiterpene cl7653, the yeast strain AM109 was used [31]. The cl7653 carrying plasmid was transformed either alone or together with a stabilised variant of HMG2(K6R) to increase substrate availability [33]. As seen in Fig. 3a, cl9841 is an active monoterpene synthase enzyme producing a range of monoterpenes with the most prominent being camphene (52.55 %), β-myrcene (11.01 %) and limonene (10.44 %) and several minor additional compounds. The cl1310 expressing cells did not produce any compounds. The cl7653 enzyme was active and less promiscuous than cl9841, producing mainly caryophyllene (86.4 %) and lesser amounts of α-humulene (Fig. 3b). The caryophyllene peak was additionally validated with the mass spectrum of a standard compound.

### Gas Chromatography/Mass Spectrometry (GC/MS) analysis of wounded leaves

GC/MS qualitative and semi-quantitative analysis was carried out for collected leaves, 4 hours (h) after their mechanical wounding. Unwounded leaves were also collected. The results are given in Tables 4 and 5. In particular, each leaf extract component is cited and accompanied by its retention time and a peak area percentage calculated by the GC/MS Solution software. Table 4 shows the common compounds detected both in unwounded and wounded leaves. Wounding can significantly affect the concentration of substances which are mainly aldehydes, ketones and alcohols.

**Table 4:** Common compounds in unwounded and wounded leaves as resulted from GC/MS analysis

Retention time (min)	Area (%)		Name
	Unwounded leaf	Wounded leaf	
4.02	22.20	19.20	(*E*)- 2-Hexenal
4.79	0.12	0.12	Sorbaldehyde
10.49	1.93	0.72	7-Methyl-4-octanol
11.17	1.46	2.00	Phenethyl alcohol
18.13	0.18	0.21	4,6-Dimethyldodecane
18.57	0.35	0.78	(*E,E*)-2,4-Decadienal
19.21	0.19	0.22	*n*-Tridecanol
19.54	1.24	0.99	2,4-Dodecadien-1-al
26.48	0.76	0.43	*trans*-beta-Ionone
28.84	0.49	0.43	2,6,11-Trimethyldodecane
29.26	0.29	0.28	2-Hexyl-1-decanol
29.66	0.28	0.43	Octadecyl chloride
35.26	6.57	2.58	Myristaldehyde
39.87	0.32	1.04	Phytone
40.51	0.85	0.63	1,2-Benzenedicarboxylic acid, bis (2-methylpropyl) ester
45.04	0.65	0.48	*n*-Eicosane
47.33	0.86	3.95	*cis*-9-Octadecen-1-ol
48.67	22.41	19.49	Phytol
55.25	0.86	0.45	5-Methyl-5-(4,8,12-trimethyltridecyl) dihydro-2(3H)-furanone
59.34	6.30	7.53	*n*-Tetracosane
60.41	1.25	1.63	1,2-Benzenedicarboxylic acid, 1,2-bis(2-ethylhexyl) ester
67.21	2.48	2.26	(*Z)*-13-Docosenamide
68.26	7.75	3.55	Squalene
69.90	4.84	2.74	Benzenamine, 4-(1,1,3,3-tetramethylbutyl)-N-[4-(1,1,3,3-tetramethylbutyl) phenyl]-

Wounded leaves were harvested 4 h after wounding. Unwounded leaves were harvested in the same time

**Table 5:** GC/MS analysis of compounds induced in wounded leaves

Retention time (min)	Area (%)	Name
6.74	1.18	2-Methyl-3-octanone
10.82	0.21	*n*-Nonanal
16.57	0.35	4-Oxononanal
17.30	0.07	*(E*)-2-Decenal
22.13	0.35	Pyran-2-one <2H-, 6-[hex-(3*Z*)-enyl] tetrahydro->
22.93	0.73	(*Z*)-Jasmone
23.83	0.22	(*E*)-Caryophyllene
28.57	0.10	3,9-Diethyl-6-tridecanol
31.11	0.11	*n*-Hexadecane
34.86	0.11	*n*-Heptadecane
41.79	0.39	*n*-Octadecane
45.86	0.25	Geranyl linalool isomer
48.14	2.28	Octadecyl vinyl ether

**Fig. 4:**
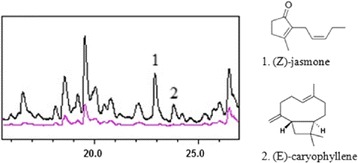
Comparative GC/MS chromatographs for unwounded (magenta line) and wounded leaves (black line). Indicated peaks 1 and 2 correspond to (*Z*)-jasmone and (*E*)-caryophyllene respectively

The substances induced in wounded leaves are included in Table 5. What is interesting to point out is that the majority of the compounds with peak area over 0.2 % are aldehydes previously associated with wounding responses. (*E*)-caryophyllene is also induced by the wounding procedure as it was detected in the GC/MS chromatogram of the wounded leaves at 23.83 min. From Fig. 4, it is obvious that the characteristic peaks of (*Z*)-jasmone and (*E*)-caryophyllene occur only in the chromatogram of the wounded leaves. The presence of (*E*)-caryophyllene and geranyl linalool in wounded leaves was also validated by comparing both the peak retention times and mass spectra between the unknown samples and standards. The mass spectra of the later as acquired from unknown samples are illustrated in figures in Additional file 5: Figure S4 and Additional files 6: Figure S5 respectiverly, with typical mass fragments at m/z 133, 93, 69 for (*E*)-caryophyllene and 69, 81, 41 for geranyl linalool respectively.

### Wounding and *TPS* expression

Since GC/MS analysis conducted in wounded leaves showed the rise in the synthesis of the sesquiterpene (*E*)-caryophyllene, the expression of cl7653 identified as caryophyllene synthase, was studied in real-time PCR experiments. Given that no monoterpene compounds were detected in the extractions of wounded leaves, the expression of cl9841 was not studied. What was studied was the expression of cl1310 - putative *KS*, of unigene2314 - putative *DXS2* and of cl1634 - putative *HMGR1* along with the *allene oxide cyclase* (*AOC*) gene, a gene involved in the formation of jasmonic acid (JA) and quickly induced by wounding in tomato leaves [34, 35]. Primers were designed to amplify the specific contigs of each of the clusters cl7653 and cl1310 (as analyzed above) while for cluster cl1634 primers were designed to amplify a common region of all three alleles.

**Fig. 5:**
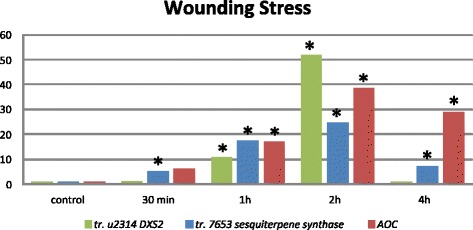
*S. elaeagnifolium* transcript abundance in wounded leaves versus the control-unwounded leaves. Control corresponds to unwounded leaves collected from three plants; 30 min, 1 h, 2 h, 4 h are the time points when wounded leaves from four plants were collected. Each plant in each time point is one biological replication. The bars represent the expression of each gene in the pooled leaves of the four biological replications-plants. A putative *S. elaeagnifolium AOC* gene is used as the wounding monitoring gene. *S. elaeagnifolium EF1a* was used as reference. Asterisks indicate statistically significant differences between the control and the samples (*p < 0.05*)

As shown in Fig. 5, wounding of *S. elaeagnifolium* leaves induced the expression of the *S. elaeagnifolium AOC* homolog gene in all time points tested, providing evidence that plants undertake responses related to the wounding stress. The expression of sesquiterpene - caryophyllene synthase gene cl7653 was increased in all wounding time points compared to the control, the unwounded leaves (leaves from three independent controls-plants). The increase in the expression of cl7653 was quickly recorded at the time point 30 min after wounding. Yet the most pronounced increase in the expression of the caryophyllene synthase cl7653 was 2 h after wounding where the gene was expressed nearly 25 times significantly more than in the control. At the last time point, 4 h after wounding, the cl7653 expression was still significantly higher than the control but less than in the 1 and 2 h time points. On the other hand, the expression of unigene2314 - putative *DXS2* gene is induced later than that of cl7653. However in its peak of expression, also at the 2 h after wounding time point, unigene2314 was expressed 50 times more significantly than in the control. Interestingly its expression fell sharply reaching the same expression as in the control unwounded leaves at 4 h after wounding. The expression of putative *HMGR1* - cl1634 and *KS* - cl1310 remained unchanged (data not shown).

## Discussion

### The *S. elaeagnifolium* transcriptome

*S. elaeagnifolium* mRNA from leaves and flowers was sequenced and reads were used to build a *de novo S. elaeagnifolium* transcriptome. The inclusion of the two tissues in the RNAseq libraries provides a representative sampling of the genes expressed in this wild and unexplored *Solanum* species. From the 75,618 unigenes assembled nearly 67 % was annotated by using the NR database. The percentage of annotated transcripts is similar to the *N. benthamiana* annotated sequences based on GenBank database (68.83 %) [36]. However the proportions of *S. elaeagnifolium* unigenes that present matches with sequences in Swiss-Prot, KEGG, COG and GO databases were lower: 42 %, 39 %, 26 % and 48 % respectively. A percentage of 33 % of the unigenes had no NR hit, a number lower than the number of transcripts that remained without NR annotation in the *de novo* sequencing of sweet potato [37] but larger than those without NR annotation in chili pepper [38]. The terms “binding and catalytic activity”, “cell and cell part”, “metabolic and cell process” were the most representative of the three main GO categories of cellular component, molecular function and biological process, assigned to the assembled *S. elaeagnifolium* unigenes. Similar results were obtained from annotating the transcripts of sweet potato [37]. An interesting finding is that 20.4 % of the *S. elaeagnifolium* unigenes are classified as “response to stimulus” in the biological process GO category. Given the species tolerance to environmental stresses, genes involved are probably categorized in this percentage.

Transcript quantification estimated by RSEM software in *S. elaeagnifolium* leaves and flowers showed that most of the transcripts expressed amply are universally found to be strongly expressed in other *Solanum* databases, while some are unexpectedly abundant in *S. elaeagnifolium* leaves and flowers such as the *PR* transcripts. PR1 proteins are known defence-related proteins used by the plants in systemic acquired resistance. The high expression of these two putative *PR* genes in *S. elaeagnifolium* may imply that the plant has *a priori* constitutive defence mechanisms that make it resistant to pathogen attack. The constitutive expression of PR proteins is common in resistant cultivars [39] and has been suggested as a modern breeding goal.

### Analysis of wounded *S.elaeagnifolium* leaves

Mechanical wounding resulted in the induction of a plethora of important chemical compounds in *S. elaeagnifolium* leaves. Among them, the sesquiterpene (*E*)-caryophyllene an attractant for natural enemies that parasitize herbivores. Recently it was found that caryophyllene has an anti-bacterial activity in flowers of Arabidopsis plants [40]. Furthermore the volatile jasmone was the key compound detected in abundance in our wounded *S. elaeagnifolium* leaves indicative of the damage done. Jasmone, a product of jasmonic acid, is implicated in various aspects of plant defence [41]. This finding agrees with the rise in the expression of the JA related, wounding-monitoring *AOC* gene, recorded in wounded leaves. Apart from jasmone, the majority of the rest of the compounds found were aldehydes and ketones. *n*-Nonanal and (*E*)-2-decanal are common volatile compounds that contribute to aroma in tomato and other fruits. Interestingly, nonanal and decanal were also detected in wounded tomato leaves but their concentrations did not vary significantly from unwounded controls [42]. Nonanal was also found to be induced in damaged poplar leaves [43]. (*E*)-2-decenal from *Ailanthus altissima* was found to have activity towards nematodes of the *Meloidogyne* genus [44]. (*E*)-2-decenal oil from the plant *Coriandrum sativum* was found to have anti-fungal activity as vapor against *Botrytis*, *Alternaria* and *Geotrichum* [45]. *N*-hexadecane, *n*-heptadecane and *n*-octadecane are also volatile compounds detected in many plants [46, 47].

Geranyl linalool is a diterpene alcohol produced by GGPP via the MEP pathway. Geranyl linalool further produces the volatile (*E,E*)-4,8,12-trimethyltrideca-1,3,7,11-tetraene (TMTT), an insect-induced terpene that is released from plants such as Arabidopsis and tomato under the attack of herbivores [48, 49]. It was shown that in tomato both geranyl linalool and TMTT are induced by JA treatment [50].

### Terpene related genes and their expression after wounding

As a tentative to isolate genes related to terpene biosynthesis from *S. elaeagnifolium* we retrieved putative *TPS* genes using BLAST algorithms. Cl7653 was the one most expressed putative *TPS*. Its deduced amino acid sequence shares high homology (79 %) with sesquiterpene synthase *Lycopersicum hirsutum2* (SSTLH2) protein from *S. habrochaites* that catalyzes the formation of germacrene D [51]. Cl7653 is similar to tomato sesquiterpene synthases genes *TPS9*, *TPS10*, *TPS12* and two *SSTLH* from *S. habrochaites*. TPS12 synthesizes *β*-caryophyllene and α-humulene [52]. Cl7653 is wounding-responsive in *S.elaeagnifolium* leaves. Its induced expression that peaked 2 h after wounding suggests that probably this gene is involved in the defence plant system. In agreement with this increase in cl7653 transcriptomic activity, 2 h after wounding, CG/MS analysis has certified the increase in (*E*)-caryophyllene emission in wounded leaves 2 h after the cl7653 transcription peak, making highly probable that cl7653 is actually the gene responsible for the production of caryophyllene in *S. elaeagnifolium*. The particular finding is in accordance with the results in yeast cells showing that the expression of cl7653 produces (*E*)-caryophyllene. A (*E*)-caryophyllene synthase in maize was increased after attack in roots by *Diabrotica virgifera* larvae and in leaves by *Spodoptera littoralis*. The gene has a breeding value since it is low expressed in North American maize cultivars while it is higher in European ones [53]. Cotton roots that have been treated with methyl-jasmonate also show an increase in a *TPS* that produces (*E*)-caryophyllene indicative of the involvement of this gene in herbivory attack defense systems [54]. A similar wounding-responsive profile is also adopted by the *S. elaeagnifolium* putative *DXS* gene - unigene2314; its expression is even more pronounced than *TPS* cl7653 but it drops more drastically as the time after wounding proceeds. *DXS* is a gene involved in the MEP pathway, residing in the chloroplasts normally involved in monoterpenoid production (i.e. camphene) and diterpene production (i.e geranyl linalool). Normally the knockdown of *DXS2* leads to the production of more sesquiterpenes than monoterpenes in tomato [55] but work in *S. habrochaites* has shown that sesquiterpenes may also be produced in the chloroplasts [11]. There is also evidence that IPP and DMAPP may be transferred from the chloroplasts to the cytosol so that such *DXS* produced precursors are integrated to sesquiterpenes [56]. In the present wounding experiment the non-induced putative *HMGR1* combined with the high induced putative *DXS2* and *TPS12* (cl7653) showed that probably in *S. elaeagnifolium* the MEP pathway provides more terpenoid precursors for the production of sesquiterpenes than the MVA pathway.

## Conclusions

*S. elaeagnifolium* is an important invasive species and a serious threat for crops in several areas around the world. Here, a leaves and flowers transcriptome was generated by next-generation sequencing, identifying 75,618 unigenes with mean length of 1,082 nt. Analysis of transcript abundance showed several genes associated with stress resistance. Some of them such as *PR-like* genes were uniquely abundant to *S. elaeagnifolium*. Leaf wounding experiments showed induction of numerous aldehydes, most of them known to participate in biotic stress resistance. Additionally, two terpenes, (*E*)-caryophyllene and geranyl linalool were detected in wounded tissues. Analysis of identified full length *TPS* genes identified a caryophyllene synthase and a camphene synthase. Real-time PCR confirmed the up-regulation of the caryophyllene synthase upon wounding and putative *DXS2* which could relate to geranyl linalool and (*E*)-caryophyllene.

## Methods

### RNA sequencing and annotation of unigenes

For RNA-seq libraries total RNA was extracted from leaves and flowers of at least four *S.elaeagnifolium* open-field plants. mRNA was isolated using the FastTrack MAG mRNA isolation kit (Life technologies, Carlsbad, CA, USA). Mixed with the fragmentation buffer, mRNA was fragmented and the cDNA synthesized using the mRNA fragments as templates. Short fragments were purified and resolved in elution buffer, for end reparation and single nucleotide A addition. After that, the short fragments were connected with adapters. The suitable fragments were selected for the PCR amplification as templates. During the quality control steps, Agilent 2100 Bioanaylzer and ABI StepOnePlus Real-Time PCR System were used in quantification and qualification of the sample library. Finally, the library was sequenced on a Illumina HiSeq™ 2000 or other sequencer when necessary.

The raw reads produced by the sequencing were cleaned; reads with adaptors, with uknown nucleotides more than 5 %, and low quality reads were removed. Reads were *de novo* assembled into contigs using the Trinity suite [19]. The resulting contigs, called unigenes, were clustered in families and unigenes were divided into two classes. One is clusters, with the prefix cl, containing several unigenes whith similarity between them more than 70 %. The other is singletons with the prefix unigene. In the final step, blastx alignment (e-value <0.00001) between unigenes and protein databases like NR, Swiss-Prot, KEGG and COG was performed, and the hits with the highest similarity were used to decide the sequence direction of unigenes. If results of different databases conflicted with each other, a priority order of NR, Swiss-Prot, KEGG and COG was followed. When a unigene could not be aligned to the above databases, ESTScan [57] was used to decide the sequence direction. A summary of the pipeline is presented in figure in Additional file 7: Figure S6.

Unigenes were classified in different classes and assigned GO and COG functional annotation. Blast2GO program [58] was used to get GO annotation of unigenes based on NR. After GO annotation, WEGO software [59] was used to do GO functional classification for all unigenes.

Transcript quantification was estimated from RNA-seq data using the RSEM software package. Unigenes were used as reference to estimate the abundance of expression based on the paired-end RNA-Seq data using the standard instructions and parameters as described in http://deweylab.biostat.wisc.edu/rsem/README.html.

### Bioinformatics analysis for identifying *S. elaeagnifolium* terpene-related genes

A dataset of expressed terpenes-related genes from tomato and other *Solanum* species, was formed using sequences retrieved from the NCBI protein database. Terpene-related genes included the genes involved in the production of proteins of the MVA and MEP pathways and TPSs. The proteins of this dataset were used as queries in BLAST searches (tblastn algorithm, e-value 10^−8^) against our *S. elaeagnifolium* unigenes database. Several unigenes (both in clusters and singletons) were retrieved similar to *Solanum* genes. Emphasis was given in our study on five genes (unigenes), two encoding key proteins of the MVA and MEP pathways and three, important for the biosynthesis of terpenes, TPS proteins.

### Wounding and expression by real-time PCR

*S. elaeagnifolium* seeds were collected from open-field plants fruits grown in the Aristotle university farm. The seeds were left to dry and then placed in water for 5 days in the dark. The emerging plantlets were then sown in small pots in the greenhouse until their transplantation in larger pots under stable temperature conditions. For the mechanical wounding experiment, leaves from plantlets with up to six to eight true leaves were cut with scissors and were collected 30 min (time point 1), 1 h (time point 2), 2 h (time point 3) and 4 h (time point 4) after wounding. Four plants were wounded and their leaves were collected in each time point while leaves were collected also from three control plants (unwounded, time point 0). All leaves, wounded and unwounded, were immediately frozen in liquid nitrogen and stored at -80 °C. Total RNA was extracted using the TRIzol Reagent according to the manufacturer’s protocol (Life technologies). The quantity and quality of the extracted total RNA was assessed by gel electrophoresis. First strand cDNA synthesis was carried out using as template 1 μg of each extracted total RNA, 0.5 mM dNTPs, 1× First-strand buffer, 10 mM DTT, 200 units (U) SuperScript II reverse transcriptase (Life technologies) and 250 ng random hexamers in 20 μl total volume, according to the manufacturer’s protocol.

Relative quantitative expression analysis was performed using primers (see table in Additional file 8: Table S2) specifically designed for real-time PCR amplification and -where possible- in two different exons based on information retrieved from the tomato gene orthologs. Real-time RT-PCR reactions were performed in a Rotor Gene 6000 (Qiagen) realtime PCR system. The reactions were performed in 1× KAPA SYBR® FAST Universal 2× qPCR master mix (Κapa Biosystems, Wilmington, MA, USA) containing 0.5 μM of each primer. The template was 1 μl of cDNA dilutions synthesized as described above. The cycling parameters were incubation at 95 °C for 2 min, followed by 30 or 35 cycles of 95 °C for 5 s, 60 °C for 20 s, 72 °C for 5 s, and a final extension step of 10 min at 72 °C. For the identification of the PCR products, a melting curve analysis was performed from 65 to 95 °C with each observation taken every 0.2 °C and a 5 s hold between observations. The *AOC* gene was used as a wounding monitoring control gene. The *S. elaeagnifolium* putative *AOC* ortholog (unigene23589) was identified using BLAST algorithms. The eukaryotic translation *elongation factor-1a* (*EF1a*) gene was used as reference; using BLAST algorithms the putative *EF1a* gene (cl630) was retrieved from *S. elaeagnifolium* unigenes bearing high similarity with tomato [NCBI: NM_001247106.1] and potato [GenBank: AB061263.1] *EF1a* genes (for *AOC* and *EF1a* primers see table in Additional file 8: Table S2). Two technical replications were performed for each biological replication i.e. each wounded plant. Relative quantitation and statistical analysis were performed using the REST software [60].

### GC/MS analysis

Samples from wounded and unwounded leaves (stored at -80 °C) were also used for GC/MS analysis. Leaves were collected 4 h after wounding. Leaves samples were pulverized in a mortar under liquid nitrogen. About 1 g of fine powder was extracted with 4 ml of a hexane:diethyl ether (90:10 v/v) mixture using vortex for 1 min. The mixture was then centrifuged for 2 min at 20,238 g. After the phase separation, the supernatant liquid was collected, dried with anhydrous sodium sulfate and filtered through a PTFE syringe filter (0.45 μm × 25 mm). The resultant extract was then concentrated to a final volume of 0.2 ml under nitrogen purge prior to GC/MS analysis. Leaf extracts were analyzed using a GC-2010 Plus Shimadzu gas chromatograph equipped with a GCMS-QP2010 Ultra gas chromatograph mass spectrometer, and a MEGA-5MS capillary column (30 m × 0.25 mm, 0.25 μm film thickness), in the splitless mode. The temperature of injector and detector was 250 °C and 300 °C respectively. The oven temperature was slowly increased with a rate of 3 °C/min from 60 °C up to 240 °C and maintained at this temperature for 5 min to equilibrate. Then the temperature was raised with 10 °C/min at 290 °C and kept isothermally for 10 min in order to elute compounds with higher boiling points. The carrier gas used for the analysis was helium at a flow rate of 1.3 ml/min. Mass spectra were acquired in a scan mode, while qualitative analysis was based on library search by using the following mass spectral libraries: FFNSC GC/MS Ver. 1.3 and Metabolite Component Database by Shimadzu, Wiley 7, NIST 11 and NIST 11 s.

### Terpene production and analysis from yeast cells

Yeast strains grown on selective plates media were used to inoculate 5 ml liquid cultures incubated overnight at 30 °C. For sesquiterpene analysis, an overlay of 500 μl dodecane (1:10 v/v) was then added and the mixture was incubated for additional 2 days at 30 °C with shaking. Dodecane phase was isolated, centrifuged (20,238 g, 2 min) and about 100 μl were removed to be injected for GC analysis. Dodecane (≥99 %) and *n*-hexane (≥99 %) used for yeast extraction and standard preparation were both purchased from Sigma-Aldrich (St. Louis, MO, USA). Dodecane extracts from yeast cultures were analyzed using a GC-2010 Plus Shimadzu gas chromatograph-mass spectrometer as above. The temperature of injector and detector was 230 °C and 270 °C respectively. The oven temperature was initially held at 60 °C for 3 min and subsequently increased up to 190 °C with a rate of 10 °C/min. Then the temperature was slowly raised with 3 °C/min at 230 °C and kept isothermally for 20 min. The carrier gas used for the analysis was helium at a flow rate of 1.66 ml/min. For the qualitative and quantitative analysis, stock solution of caryophyllene in hexane was made and a calibration curve was drawn from the prepared working solutions. Monoterpene production from yeast cells was carried out as above, using an overlay of diisononyl phthalate (≥99 %) purchased from Sigma-Aldrich. The extracts were analysed by means of GC/MS with temperature of injector and detector at 230 °C and 300 °C correspondingly. The oven temperature was increased with 3 °C/min from 60 °C to 240 °C, maintained at this temperature for 5 min to equilibrate and subsequently elevated with a rate of 10 °C/min with a final isotherm at 290 °C for 5 min.

### Availability of data

Illumina Hiseq 2000 raw transcriptome sequences are available at NCBI SRA database under the experiment accession number SRX1030234.

## Additional files

Additional file 1: Figure S1. The length distribution of assembled contigs and unigenes. On the x- axis the contigs and unigenes length in nucleotides (nt) and on y- axis the number of contigs and unigenes of each length. Additional file 2: Figure S2. GO category assignment for *S. elaeagnifolium* unigenes. Unigenes were categorized in the three categories of molecular function, cellular component and biological process. Most abundant GO-terms are cell in cellular component category, metabolic process in biological process category and binding in molecular function category. Additional file 3: Figure S3. Classification of *S. elaeagnifolium* unigenes into COG functional categories. Additional file 4: Table S1.
*S. elaeagnifolium* unigenes that bear similarity with annotated Solanaceae *TPS* genes. In some cases more than one unigenes have a hit on the same *TPS* in different sites. In the last column the FPKM values of unigenes are reported. The *TPSs* analyzed in the study are highlighted in bold. Additional file 5: Figure S4. Mass spectrum of (*E*)-caryophyllene detected in wounded leaves. Additional file 6: Figure S5. Mass spectrum of geranyl linalool detected in wounded leaves. Additional file 7: Figure S6. The pipeline used in the assembly process of *S. elaeagnifolium* mRNA reads. After the assembly of reads into contigs and the mapping of reads again into contigs, contigs were assembled in clusters of unigenes (prefix cl). All contigs not included in clusters remained as singletons (prefix unigene). Additional file 8: Table S2. Primers used in the experiments and their sequences.

## Abbreviations

TPS: Terpene synthase
MVA: Mevalonate
MEP: Methylerythritol phosphate
DXS2: 1-deoxy-D-xylulose-5-phosphate synthase 2
PVY: Potato virus Y
TYLCV: Tomato yellow leaf curl virus
DMAPP: Dimethylallyl diphosphate
GPP: Geranyl diphosphate
FPP: Farnesyl diphosphate
GGPP: Geranylgeranyl diphosphate
NPP: Neryl diphosphate
GA: Gibberellins
ABA: Abscisic acid
ESTs: Expressed sequence tags
SSR: Simple sequence repeat
nt: Nucleotides
BLAST: Basic local alignment search tool
NR: Non-redundant
KEGG: Kyoto encyclopedia of genes and genomes
GO: Gene ontology
COG: Clusters of orthologous groups
CDS: Coding DNA sequence
FPKM: Fragments per kilobase of exon per million fragments mapped
Rubisco: Ribulose-1,5-bisphosphate carboxylase/oxygenase
SAMDC: S-adenosylmethionine decarboxylase
MTs: Metallothioneins
EC: Enzyme commission number
GAP: d-glyceraldehyde-3-phosphate
DHAP: Dihydroxyacetone phosphate
FBP: Fructose-1,6-bisphosphate
PR: Pathogenesis-related
HMGR: 3-hydroxy-3-methylglutaryl-coenzyme A reductase
AACT: Acetoacetyl CoA thiolase
MCT: 2-C-methyl-d-erythritol 4-phosphate cytidylyl-transferase
FPPS: Farnesyl pyrophosphate synthase
GGPPS: Geranylgeranyl pyrophosphate synthase
CPT: Cis-prenyltransferase
TFGD: Tomato functional genomics database
RNA-seq: RNA-sequencing
CAHS: Caryophyllene/α-humulene synthase
KS: *ent*-kaurene synthase
GC/MS: Gas Chromatography/Mass Spectrometry
AOC: Allene oxide cyclase
TMTT: (*E,E*)-4,8,12-trimethyltrideca-1,3,7,11-tetraene
SSTLH2: Sesquiterpene synthase *Lycopersicum hirsutum2*
EF1a: Elongation factor-1a
