# Supplementary figures and images for: Use of the de novo transcriptome analysis of silver-leaf nightshade (Solanum elaeagnifolium) to identify gene expression changes associated with wounding and terpene biosynthesis
Source: BMC Genomics. 2015 Jul 7;16(1):504. doi: 10.1186/s12864-015-1738-3 (PMC4492009; doi:10.1186/s12864-015-1738-3)

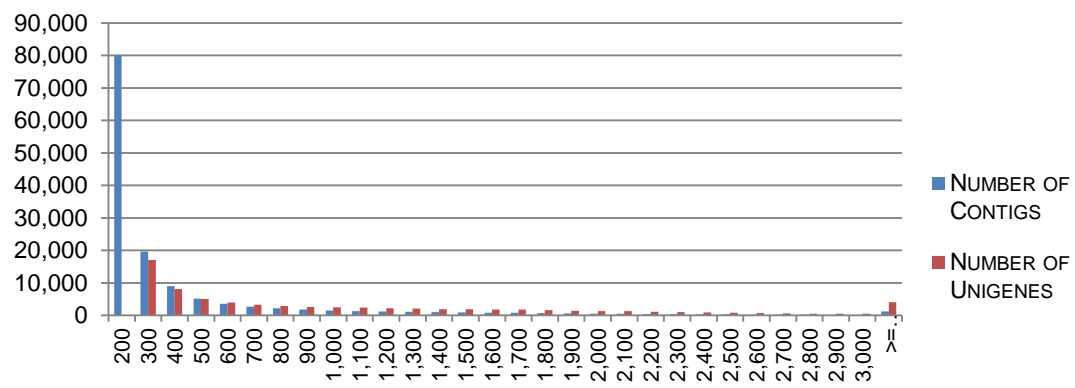

Supplement: Additional file 1: Figure S1. — The length distribution of assembled contigs and unigenes. On the x- axis the contigs and unigenes length in nucleotides (nt) and on y- axis the number of contigs and unigenes of each length. [file 12864_2015_1738_MOESM1_ESM.pdf]

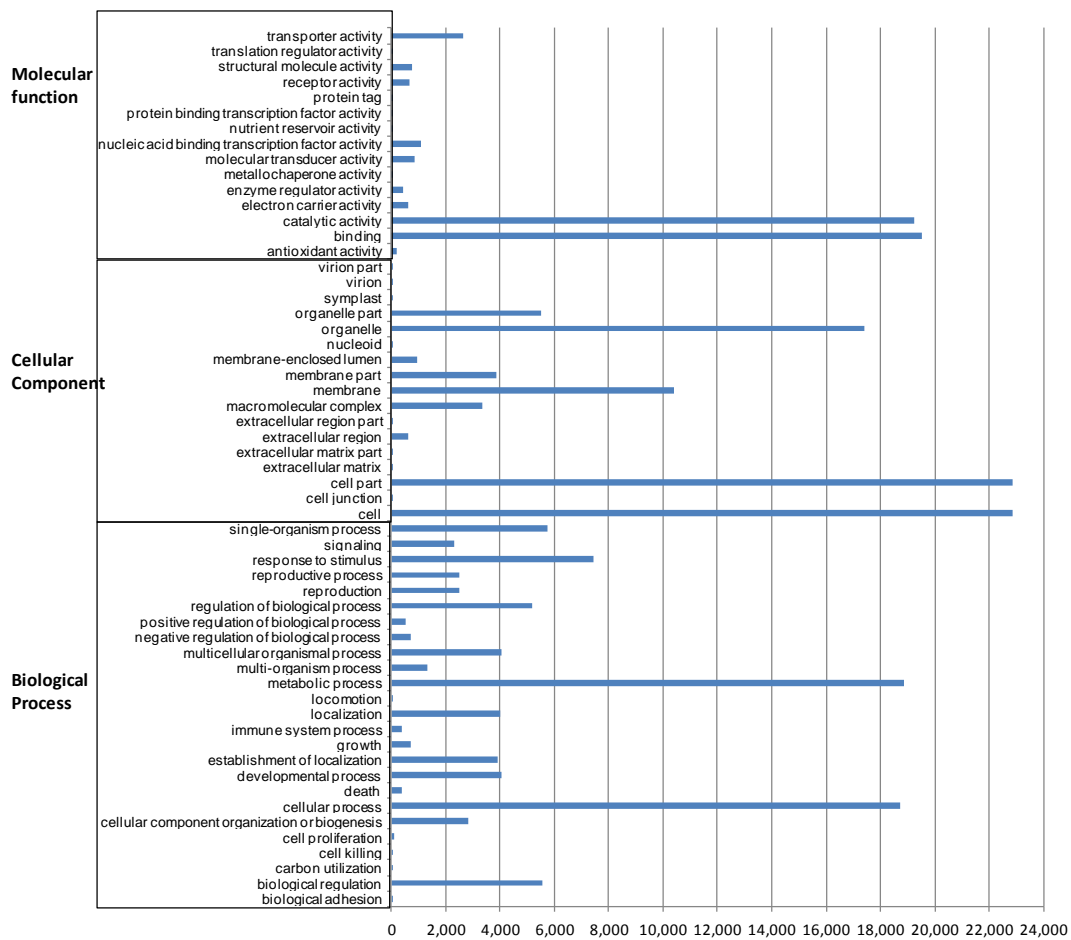

Supplement: Additional file 2: Figure S2. — GO category assignment for S. elaeagnifolium unigenes. Unigenes were categorized in the three categories of molecular function, cellular component and biological process. Most abundant GO-terms are cell in cellular component category, metabolic process in biological process category and binding in molecular function category. [file 12864_2015_1738_MOESM2_ESM.pdf]

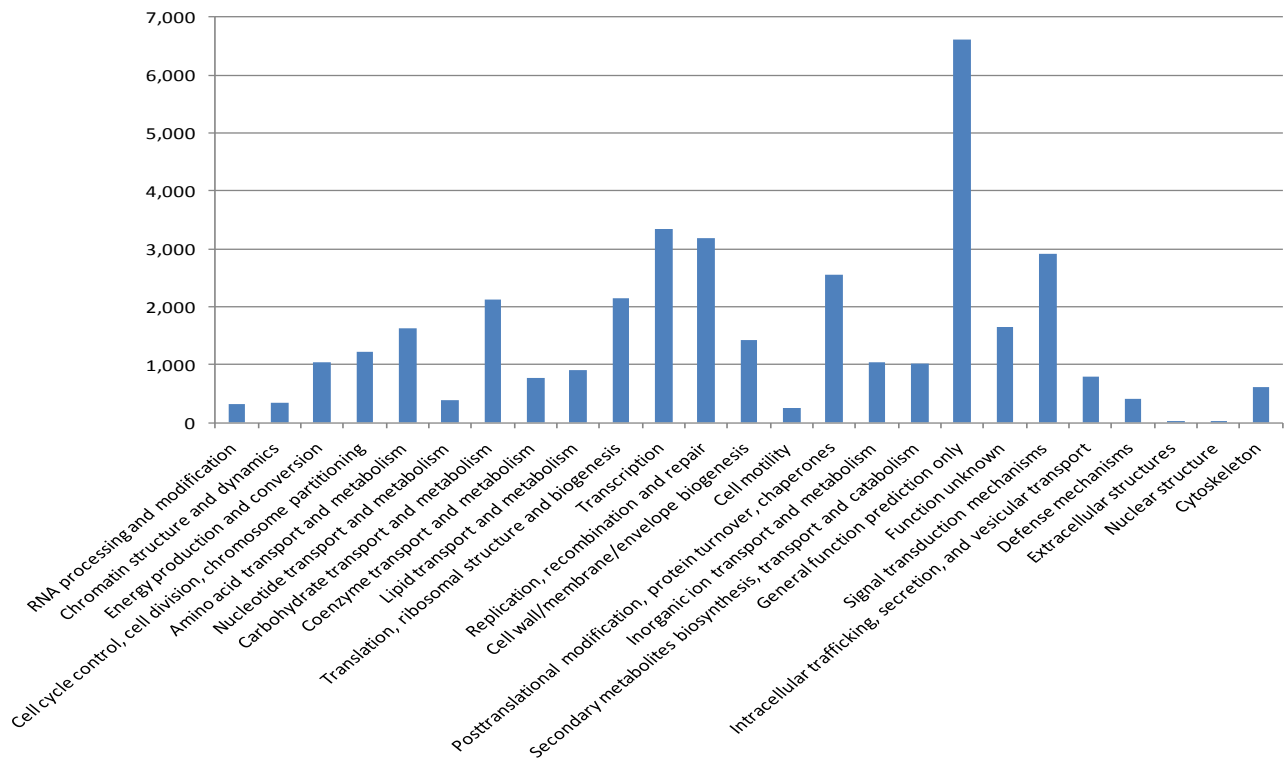

Supplement: Additional file 3: Figure S3. — Classification of S. elaeagnifolium unigenes into COG functional categories. [file 12864_2015_1738_MOESM3_ESM.pdf]

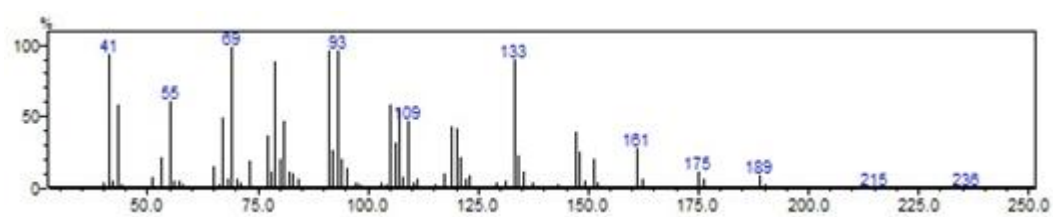

Supplement: Additional file 5: Figure S4. — Mass spectrum of (E)-caryophyllene detected in wounded leaves. [file 12864_2015_1738_MOESM5_ESM.pdf]

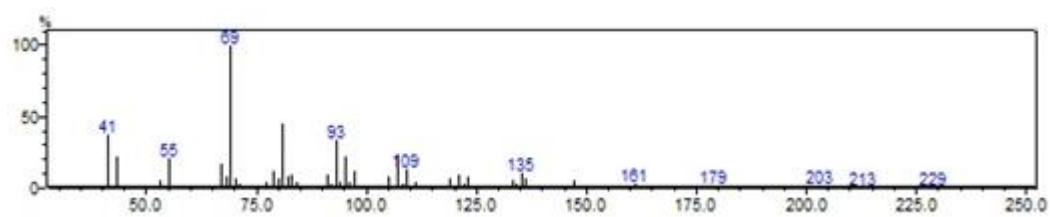

Supplement: Additional file 6: Figure S5. — Mass spectrum of geranyl linalool detected in wounded leaves. [file 12864_2015_1738_MOESM6_ESM.pdf]

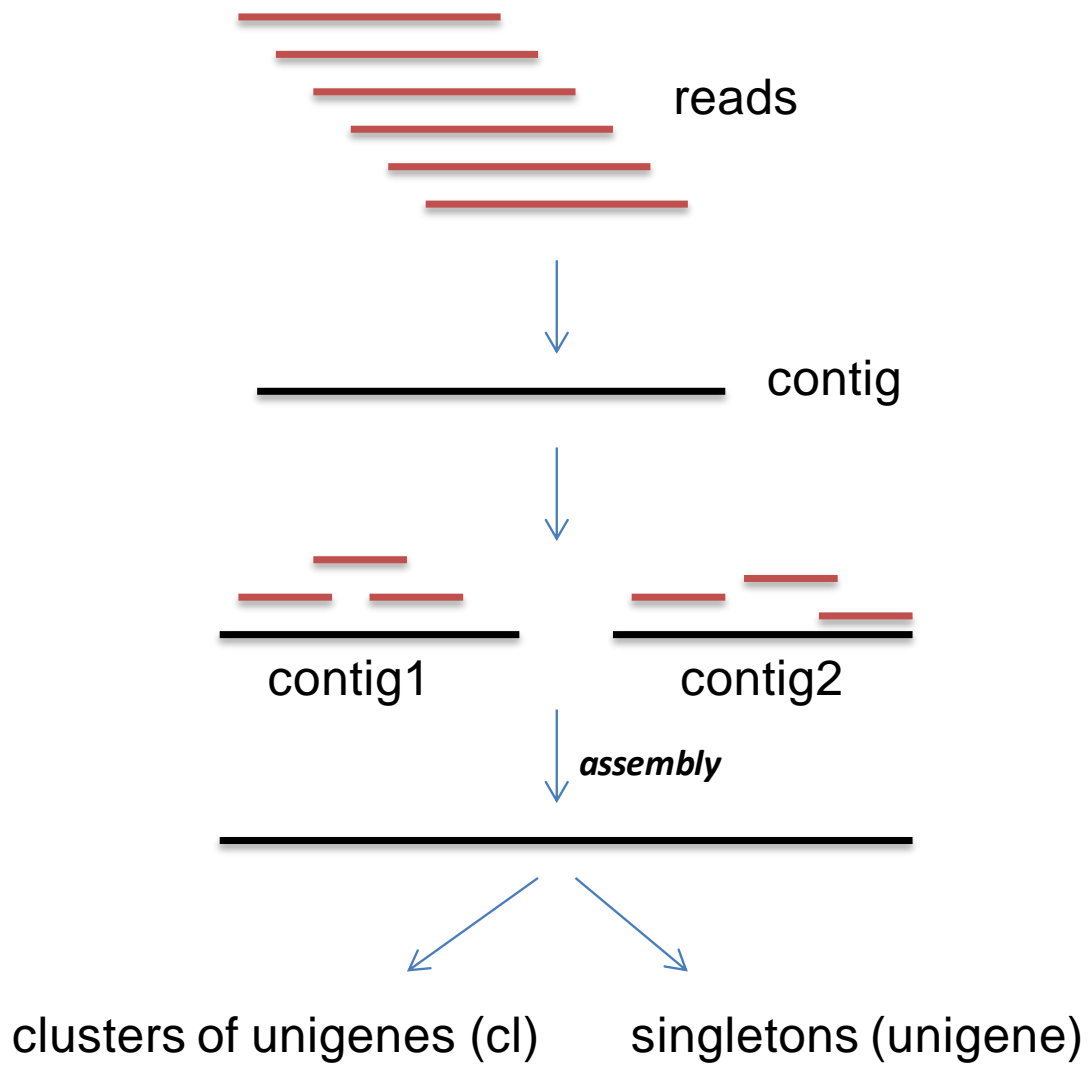

Supplement: Additional file 7: Figure S6. — The pipeline used in the assembly process of S. elaeagnifolium mRNA reads. After the assembly of reads into contigs and the mapping of reads again into contigs, contigs were assembled in clusters of unigenes (prefix cl). All contigs not included in clusters remained as singletons (prefix unigene). [file 12864_2015_1738_MOESM7_ESM.pdf]
