# Supplementary material for: Use of the de novo transcriptome analysis of silver-leaf nightshade (Solanum elaeagnifolium) to identify gene expression changes associated with wounding and terpene biosynthesis
Source: BMC Genomics. 2015 Jul 7;16(1):504. doi: 10.1186/s12864-015-1738-3 (PMC4492009; doi:10.1186/s12864-015-1738-3)
Supplement: Additional file 4: Table S1. — S. elaeagnifolium unigenes that bear similarity with annotated Solanaceae TPS genes. In some cases more than one unigenes have a hit on the same TPS in different sites. In the last column the FPKM values of unigenes are reported. The TPSs analyzed in the study are highlighted in bold. [file 12864_2015_1738_MOESM4_ESM.pdf]

***S.elaeagnifolium* transcript**	**Length (nt)**	**Top annotated hit (Species)**	**FPKM**
**cl9841.contig1**	**2,040**	**TPS3 (SOLLC)**	**7.2**
**cl7653.contig9**	**2,143**	**SSTLH2 (SOLHA)**	**22.9**
**cl1310.contig14**	**2,801**	**TPS24 (SOLLC)**	**4.6**
cl3422.contig4	1,768	TPS25 (SOLLC)	5.1
cl3115.contig1	1,385	TPS37 (SOLLC)	8.6
cl4017.contig3	2,266	TPS38 (SOLLC)	6.2
cl3304.contig2	2,364	TPS40 (SOLLC)	0.8
cl10191.contig1<br>unigene2293<br>unigene37654<br>unigene29155	451<br>442<br>263<br>369	TPS7 (SOLLC)	1.6<br>1.1<br>0.7<br>5.0
cl9158.contig1	210	TPS5 - MTS1 (SOLLC)	0.9
unigene2549<br>unigene32546	501<br>291	TPS36 (SOLLC)	0.6<br>1.6
unigene9042<br>cl3483.contig2<br>unigene36372	492<br>555<br>266	TPS17 (SOLHA)	3.8<br>5.1<br>1.3
unigene41633	201	TPS15b (SOLHA)	0.7
unigene38531<br>unigene25181<br>unigene10135	202<br>283<br>281	TPS25 (SOLLC)	0.6<br>1.1<br>1.7
unigene34833<br>unigene2853	256<br>219	TPS28 (SOLLC)	1.4<br>0.9
