# Supplementary material for: Use of the de novo transcriptome analysis of silver-leaf nightshade (Solanum elaeagnifolium) to identify gene expression changes associated with wounding and terpene biosynthesis
Source: BMC Genomics. 2015 Jul 7;16(1):504. doi: 10.1186/s12864-015-1738-3 (PMC4492009; doi:10.1186/s12864-015-1738-3)
Supplement: Additional file 8: Table S2. — Primers used in the experiments and their sequences. [file 12864_2015_1738_MOESM8_ESM.pdf]

Primer name	Primer sequence 5’→3’
FOR-cl7653 (TPS)	GCTTGGTGGGTATGGAGGAAT
REV-cl7653 (TPS)	GTTTGTGTATCCATCTTCATCTTG
FOR-cl1310 (TPS)	GTTGGACCCAAGCTCTCAGAT
REV-cl1310 (TPS)	GTACACTGCCCTCTCCTTGC
FOR-cl1634 (HMGR1)	CTTCTCGGTTTCTTTGGGATTG
REV-cl1634 (HMGR1)	CTCTGCAACGCCTCTTTACG
FOR-unigene2314 (DXS2)	GCATCTCTGCTGGTCTTGGT
REV-unigene2314 (DXS2)	GAGTTGCAGGGCCATCTAAG
FOR-cl630 (EF1a)	CTCCAAGGCTAGGTATGATGA
REV-cl630 (EF1a)	ACAGTTCCAATACCACCAATCT
FOR-unigene23589 (AOC)	CTGCTTATCTTCGATTGAGCC
REV-unigene23589 (AOC)	CTTGATCAGAATGCAGAGTCC
